# Protein kinases of the human malaria parasite *Plasmodium falciparum*: the kinome of a divergent eukaryote

**DOI:** 10.1186/1471-2164-5-79

**Published:** 2004-10-12

**Authors:** Pauline Ward, Leila Equinet, Jeremy Packer, Christian Doerig

**Affiliations:** 1 Wellcome Centre for Molecular Parasitology, University of Glasgow, 56 Dumbarton Road, Glasgow G11 6NU, Scotland, UK; 2 INSERM U609, Wellcome Centre for Molecular Parasitology, University of Glasgow, 56 Dumbarton Road, Glasgow G11 6NU, Scotland, UK; 3 Division of Advanced Technologies, Abbott Laboratories, 100 Abbott Park Road, Abbott Park, IL 60064, USA

## Abstract

**Background:**

Malaria, caused by the parasitic protist *Plasmodium falciparum*, represents a major public health problem in the developing world. The *P. falciparum *genome has been sequenced, which provides new opportunities for the identification of novel drug targets. Eukaryotic protein kinases (ePKs) form a large family of enzymes with crucial roles in most cellular processes; hence malarial ePKS represent potential drug targets. We report an exhaustive analysis of the *P. falciparum *genomic database (PlasmoDB) aimed at identifying and classifying all ePKs in this organism.

**Results:**

Using a variety of bioinformatics tools, we identified 65 malarial ePK sequences and constructed a phylogenetic tree to position these sequences relative to the seven established ePK groups. Predominant features of the tree were: (i) that several malarial sequences did not cluster within any of the known ePK groups; (ii) that the CMGC group, whose members are usually involved in the control of cell proliferation, had the highest number of malarial ePKs; and (iii) that no malarial ePK clustered with the tyrosine kinase (TyrK) or STE groups, pointing to the absence of three-component MAPK modules in the parasite. A novel family of 20 ePK-related sequences was identified and called FIKK, on the basis of a conserved amino acid motif. The FIKK family seems restricted to Apicomplexa, with 20 members in *P. falciparum *and just one member in some other *Apicomplexan *species.

**Conclusion:**

The considerable phylogenetic distance between Apicomplexa and other Eukaryotes is reflected by profound divergences between the kinome of malaria parasites and that of yeast or mammalian cells.

## Background

Modulation of protein phosphorylation through the antagonistic effects of protein kinases and protein phosphatases is a major regulatory mechanism of most cellular processes. Dysregulation of protein phosphorylation in human cells plays a major role in many diseases such as cancers and neurodegenerative disorders [1]. This has prompted the search for drugs targeting protein kinases, an endeavour which led in 2002 to the commercialisation of Gleevec, the first protein kinase inhibitor used as a drug for human disease [2]. Additional molecules targeting protein kinases are in clinical trial [3,4], and significant developments in this field are expected in the next few years. Some of the most devastating infectious diseases are caused by protists such as malaria parasites and trypanosomatids: hence, about half the global population lives in malarious areas, with 10% of the world population contracting the disease each year, which results in 1–3 million annual deaths. The essential role played by eukaryotic protein kinases (ePKs) in crucial cellular functions makes them attractive potential targets for drugs against such eukaryotic infectious agents [5].

Malaria parasites have a complex life cycle. Infection of human beings by *Plasmodium falciparum*, the species responsible for the lethal form of human malaria, begins with the bite of an infected *Anopheles *mosquito, which delivers sporozoites into the bloodstream. These cells establish an infection inside hepatocytes, where they undergo an intense multiplication generating several thousand merozoites, a process called exo-erythrocytic schizogony. The merozoites invade erythrocytes, where they also undergo schizogony, the process that is responsible for malaria pathogenesis. Some merozoites, however, arrest the cell cycle and differentiate into male or female gametocytes, which are infective to the mosquito. Once ingested by the insect, the gametocytes develop into gametes (which for the male cells involves three rapid rounds of cell division) and fuse into a zygote. Further development in the mosquito involves a process of sporogony, producing sporozoites that accumulate in the salivary glands and are now ready to infect a new human host (see  for information on malaria).

The observation that many parasitic ePKs display profound structural and functional divergences from their counterparts in their vertebrate hosts [5-7] suggests that specific inhibition is an attainable goal. The availability of PlasmoDB, a genomic database for *Plasmodium falciparum *[8], now permits a systematic analysis of the entire complement of ePKs encoded in the genome (the "kinome") of this pathogen, an important milestone both in our understanding of *Plasmodium *biology and in the definition of potential novel drug targets.

Prior to the genomic era, the initial classification system of Hanks and Quinn [9] distributed ePKs into four major groups:

• the cyclic-nucleotide- and calcium/phospholipid-dependent kinases (the AGC group);

• the CMGC group, constituted of the cyclin-dependent- (CDK), mitogen-activated- (MAPK), glycogen-synthase- (GSK) and CDK-like kinases;

• the calmodulin-dependent kinases (CaMK), and

• the tyrosine kinases (TyrK).

ePKs that did not clearly fit into any of these groups were placed into the OPK ("other protein kinases") group. The primary structure of all enzymes in these groups conform to the model described by Hanks, in which the catalytic domain is subdivided into eleven subdomains, which can be aligned across all groups. In addition to the "typical" ePKs, several enzymes possessing protein kinase activity, but which are unrelated (or only distantly related) to ePKs at the primary structure level, have been identified and termed "atypical protein kinases" (aPKs). These include phosphatidyl-inositol 3' kinase (PI3K), DNA-dependent protein kinase, and members of pyruvate dehydrogenase kinase family.

Exhaustive analyses of the kinome of some model organisms have now been published. The kinome of *S. cerevisiae *contains 115 ePKs [10], and the genomes of *D. melanogaster*, *C. elegans *and *H. sapiens *comprise 239, 454 and 510–520 ePK-coding genes, respectively [11-14]. On the basis of this wealth of new data, three additional major ePK groups were recognized (reviewed in [15]:

• the casein kinase 1 (CK1) group;

• the STE group, which includes many enzymes functioning in MAPK pathways, although the MAPKs themselves belong to the CMGC group (STE stands for "sterile", referring to the fact that enzymes belonging to this group were first identified in genetic analysis of yeast sterile mutants);

• the tyrosine kinase-like (TKL) group, which, as its name indicates, includes enzymes that are related to those in the TyrK group, although they are serine-threonine protein kinases.

Furthermore, a description of the 369 non-receptor serine/threonine protein kinases of the plant *Arabidopsis thaliana *has recently been published [16]. Comparative examination of this and previously available kinomes has demonstrated that members of all major ePK groups can be found in yeast, worms, insects, mammals and plants, with the exception of TyrKs, which are not found in yeast. That most TyrKs function in hormone-response receptor-linked pathways suggests that this family arose as an adaptation to the needs for intercellular communication in multicellular organisms. It has however been reported recently that a few unicellular eukaryotes (Chlamydomonoas, Entamoeba and Phytophtora) possess putative TyrK family members [17]. Despite the fact that most serine-threonine ePKs groups are found in all eukaryotes, indicating that their appearance occurred early in evolution, each of the kinomes has nevertheless its specificities. A striking feature in this respect is the considerable extension of some ePK families in some organisms but not in others. For example, yeast and *Drosophila *have 4 and 10 members of the casein kinase 1 (CK1) group respectively, whereas the *C. elegans *genome encodes 85 CK1-related genes.

**Figure 1:**
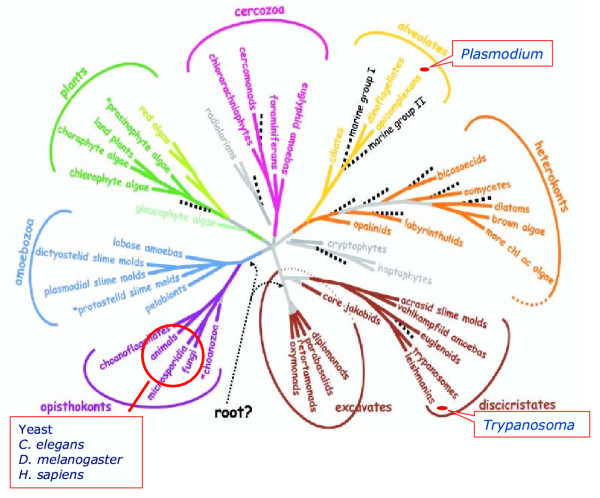
**Phylogenetic distance between malaria parasites and the organisms used as model Eukaryotes. **With the exception of the plant *Arabidopsis*, the organisms whose kinome has been characterised (yeast, worms, Drosophila and human), all belong to the *Opisthokonta *lineage, which is vastly distant from the *Alveolata *branch which include the *Apicomplexa*. Adapted from Badlauf, S (2003), with permission (Copyright 2003 AAAS).

With the exception of the plant *A. thaliana*, all eukaryotes whose kinome has been characterised, from yeast to man, belong to the Opisthokonta phylogenetic group. As depicted in Fig. 1, this lineage represents only one small branch of the eukaryotic tree. Several eukaryotes of high medical importance, such as malaria parasites (Alveolates) or trypanosomes (Discicristates), belong to phylogenetic groups that are vastly distant from both the Opisthokonta and Planta branches [18]. This is reflected by many profound peculiarities in their basic biology (see [5] for a review). Divergences from model eukaryotes can also be expected not only at the level of individual protein kinases of these organisms (as has been previously documented in a number of instances – see [5,6] for reviews), but at the level of their kinome as well. As is documented below, our analysis of the *P. falciparum *kinome confirms this prediction.

## Results and discussion

### Overview of the tree

**Figure 2:**
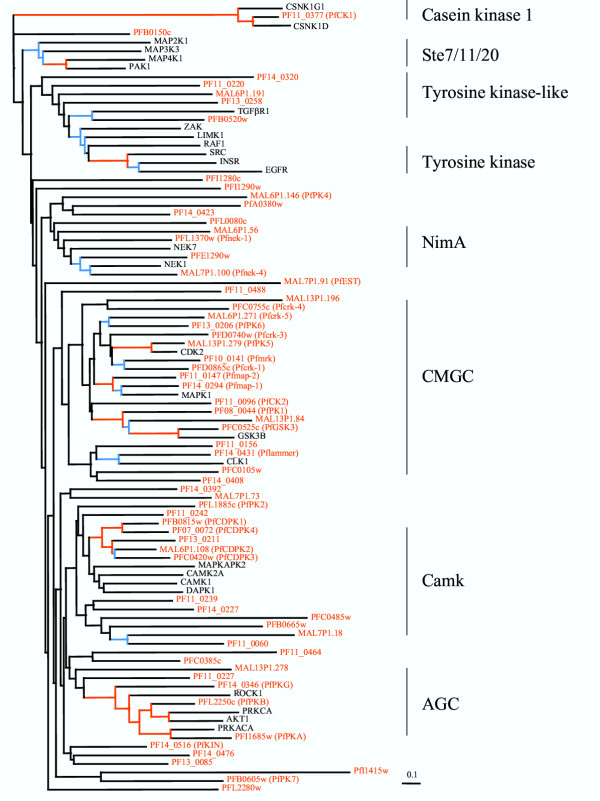
**The *P. falciparum *kinome. **Phylogenetic tree of ePKs from *P. falciparum*. The tree was compiled using conserved portions of aligned sequences using a protein distance matrix method (see Additional file 1 for the alignment). All major groupings discussed were observed in the 100 replicate bootstrap tree (not shown). Branches with bootstrap values >70 are shown in red and >40 in blue. The scale bar represents 0.1 mutational changes per residue (10 PAM units). 65 sequences from *P. falciparum *are shown (in red characters), together with representative members of major subgroups of human kinases (in black characters). The *P. falciparum *sequences are labelled with their identifier in the PlasmoDB database and, where applicable, with the published name of the enzymes. The human sequences are labeled with HUGO gene names.

65 sequences related to ePKs were retrieved from PlasmoDB and used to construct a phylogenetic tree as described in the Methods section (see Additional file 1 for the alignment). The tree of the *P. falciparum *kinome (Fig. 2) indicates that although the parasite possesses enzymes belonging to most of the major serine/threonine kinase groups, as described in the following paragraphs, several enzymes do not cluster with any of these groups.

#### CK1 group

Only one malarial kinase, the previously described PfCK1 [PF11_0377] [19], clearly falls within this group, which is vastly expanded in some other kinomes (e.g. 85 genes in *C. elegans*, see above).

#### AGC group

Five malarial kinases cluster within this group, three of which have been characterized: the cAMP-dependent PfPKA [PFI1685w] [20], the cGMP-dependent PfPKG [PF14_0346] [21], and PfPKB [PFL2250c] [22], an enzyme displaying maximal similarity to AKT/PKB. In other eukaryotes, PKB functions in the PI3K-dependent pathway; a PI3K kinase homologue is present in the *P. falciparum *genome (see below). Two additional sequences [PFC0385c and PF11_0464] form a separate cluster attached to the base of the AGC branch. There appears to be no clear member of the PKC subfamily.

#### CamK group

The main branch of the tree that contains the human CamKs also contains 13 PfePKs, which underlines the importance of calcium signalling in the parasite [23]. A tight cluster is formed by five of these enzymes, which share the overall structure of the calcium-dependent protein kinases (CDPKs) found in plants and ciliates but not in Metazoans. CDPKs are characterised by the presence of a kinase catalytic domain located on the same polypeptide as four EF-hand calcium-binding domains. Four of these enzymes have been described previously: PfCDPK1 [PFB0815w] [24], PfCDPK2 [MAL6P1.108] [25], PfCDKP3 [PFC0420w] [26] and more recently PfCDPK4 [PF07_0072]. The latter enzyme is expressed in sexual stages and was shown to be essential for development of the parasite in the mosquito, through mediating cell cycle resumption during male gametocyte exflagellation [27]. A fifth CDPK [PF13_0211], which like the four cited above possesses four EF-hand motifs, has been discovered in the present study. PF11_0242 appears to be related to CDPKs, but contains only one EF-hand motif. PfPK2 [Pfl1885c] constitutes a sister branch to the CDPK group. This enzyme was previously characterized as being related to the CamK family [28], and has no EF-hand domain. No malarial kinase clusters closely with the mammalian CamKs used to anchor the tree. Six other sequences, however, form a sister branch to the cluster that contains the CDPKs; only one of these six sequences (PF11_0239) possesses an EF-hand domain. The CamK activity described [29] as crucial for ookinete development in the mosquito vector (see below) is likely to be associated with one of the enzymes in this group.

#### CMGC group

**Figure 3:**
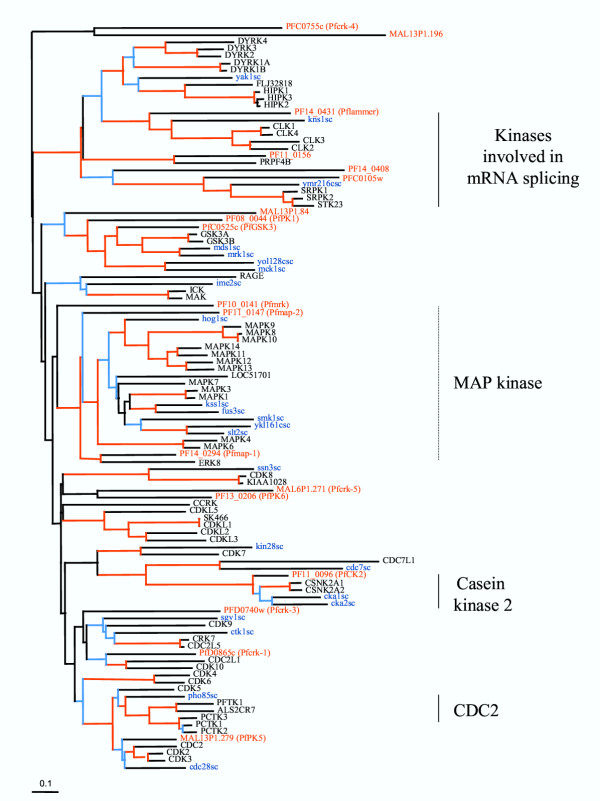
**A three-species tree of the CMGC group. **Phylogenetic tree showing members of the CMGC group of protein kinases from *P. falciparum*, yeast and human. The tree was compiled using conserved portions of aligned sequences using a protein distance matrix method; the tree shown is a consensus tree built from 100 bootstrap replicates. Branches with bootstrap values >70 are shown in red and >40 in blue. The scale bar represents 0.1 mutational changes per residue (10 PAM units). The *P. falciparum *sequences are identified by with their identifier in the PlasmoDB database and, where applicable, with the published name of the enzymes. The human sequences are labeled (black) with HUGO gene names (except for sk466, which is a numerical designation taken from Manning et al. (2002), and the yeast sequences (blue) identified according to the catalogue in Hunter and Plowman (1997).

Eighteen malarial kinases cluster within this group, which makes it the most prominent group in the *Plasmodium *kinome. Interestingly, in other eukaryotic systems a majority of CMGC kinases are involved in the control of cell proliferation and development, and their relative abundance in the *P. falciparum *kinome may reflect the variety of successive proliferative and non-proliferative stages which constitute the life cycle of malaria parasites. Six enzymes are related to the cyclin-dependent kinase family, 5 of which were identified previously (reviewed in [30]), the last one (Pfcrk-5, [MAL6P1.271]) having been discovered during the present analysis. Two previously characterised mitogen-activated protein kinases (MAPKs), Pfmap-1 [PF14_0294] [31-33] and Pfmap-2 [PF11_0147] [34], cluster together with a member of the MAPK family, as expected. Two enzymes, PfPK6 [PF13_0206] [35] and Pfcrk-4 [PFC0755c] (Equinet, Le Roch and Doerig, unpublished), display features of both CDKs and MAPKs according to BLASTP analysis. Their position either in a cluster (composed of PfPK6 and Pfcrk-5) that is intermediate between the CDK and the MAPK groups, or in a cluster (composed of Pfcrk-4 and uncharacterized MAL13P1.196) at the base of the CDK/MAPK/GSK3 branch, is consistent with these early observations. Three GSK3-related kinases, two of which [PF08_0044 and PFC0525c] have been characterised previously [36,37], form a cluster within the CMGC group. Four additional enzymes form another cluster that includes human Clk1, one of which is a previously described LAMMER-related kinase [PF14_0431] [38]. The complexity of the CMGC group, its relative importance in the *P. falciparum *kinome, and our long-standing interest in the control of cell proliferation and differentiation in the parasite, prompted us to produce a three-species comparative tree of this group (see below and Fig. 3).

#### TKL group

Five malarial enzymes appear in the vicinity of the TyrK-like group, including two [MAL6P1.191 and PFB0520w] that display maximal homology to MAPKKK-related or MLK (mixed-lineage kinases) enzymes upon BLASTP analysis. PFB0520w clusters with the TGFβ receptor (TGFβ1). The malarial sequence is much more similar to TGFβ receptors than to mammalian Raf, and furthermore, in common with TGFβ receptors, the malarial enzyme has a predicted transmembrane sequence N-terminal to the kinase domain. Mammalian TGFβ receptors assemble as heterodimers, and it remains to be seen whether the malarial enzyme forms a homodimer or has the capacity to coassemble with a mammalian subunit.

#### Absence of members of the STE and TyrK groups

No malarial protein kinase clusters with the STE7/11/20 group, which is consistent with the lack of success of earlier *in vitro *and *in silico *attempts at identifying MAPKK malarial homologues [39,40] and points to a divergent organisation of the MAPK pathways in malaria parasites (see below). It is relevant to mention here that one of the *P. falciparum *NIMA-related enzymes (see below) possesses an activation site that closely mimics that of MEK1/2. This enzyme, Pfnek-1 [PFL1370w], is able to specifically phosphorylate Pfmap-2 (but neither Pfmap-1 nor mammalian ERK2) *in vitro*, and to act in synergy with Pfmap-2 towards the phosphorylation of exogenous substrates [39]. This suggests that Pfmap-2 activity may be regulated by Pfnek-1. However, the physiological relevance of these finding remains to be demonstrated. Our tree indicates that members of the TyrK family are absent, as is the case in yeast and most (but not all) unicellular eukaryotes [17].

#### Other clusters and "orphan" kinases

Five *Plasmodium *genes form a cluster of NIMA-related sequences that includes the NIMA-related kinase Nek1. Of these five, four are recognised by BLASTP analysis as being related to the NIMA/Nek family [41], including the well characterised Pfnek-1 enzyme [39]. The fifth enzyme, MAL6P1.56, does not cluster with the NIMA-like kinases in other analyses (not shown).

Several protein kinases appear not to cluster clearly with any defined group, or to constitute small "satellite" clusters. Examples of such "orphan" kinases are (i) the cluster formed by PfKIN [PF140516], an enzyme previously described as related to the SNF1 family [42], with two uncharacterised PfPKs [PF14_0476 and PF13_0085]. This cluster is located at the base of the CamK and AGC branches, and does not strongly associate with any established ePK group (when mammalian NIM1/SNF1-like kinases were included in the phylogenetic tree no malarial kinases clustered with them (not shown)). (ii) A group of three malarial enzymes, including PfPK4 [MAL6P1.146], a previously characterised HRI kinase homologue [43], that are similar to mammalian elongation factor kinases, and form a distinct cluster associated to the NIMA group. (iii) Several sequences that are isolated at the base of major branches of the tree, indicating an absence of relatedness to established ePK groups. These include the "*P. falciparum *exported protein kinase" (PfEST, MAL7P1.91) [44], which forms an isolated branch at the base of the part of the tree containing the CMGC, CamK and AGC groups, PFL2280w, which is in a similar situation, and a group of two sequences at forming a sister cluster to the branch containing the AGC and CamK groups. One of these two sequences, PfPK7 [PFB0605w], displays relatedness to AGC and STE kinases in BLAST analysis (see below).

So far, four PfePKs have been described as appearing as "composite" enzymes displaying features from more than one established ePK family. As mentioned above, PfPK6 [PF130206] and Pfcrk-4 [PFC0755c] both display relatedness to CDKs and MAPKs, and this is confirmed by their position on the tree. The MAPKK-like activation site of Pfnek-1 [PFL1370w] has been discussed above. The fourth example is that of PfPK7 [PFB0605w], an enzyme whose C-terminal region carries a sequence which is conserved in MAPKKs but whose N-terminal region is more closely related to that of PKAs [40]. This sequence does not cluster with any well-defined group in the tree, although it associates with uncharacterized PFI1415w in a sister cluster to the major branch containing the AGC and CamK groups. Whether such "dual" enzymes represent common ancestors to subsequently divergent families which have been conserved in the evolution of the Apicomplexan lineage, or whether they arose from domain shuffling between existing kinase genes, remains to be elucidated. It is possible that additional such "composite" enzymes will be identified, particularly among the PfPKs which do not associate with well defined PK groups.

### A three-species comparison of CMGC kinases

Because of the large number of CMGC-group kinases found in the *P. falciparum *genome, we carried out a more thorough analysis in which the 18 malarial kinases belonging to this group were compared with comprehensive sets of related kinases from the yeast and human genomes (Fig. 3). The phylogenetic tree was constructed in a similar way to that in Figure 2. 152 amino acid positions from the alignment were used in the construction of the tree.

#### Evidence for absence of typical 3-component MAPK pathways

In this analysis, both *P. falciparum *kinases (Pfmap-1 and Pfmap-2) previously reported as belonging to the ERK family clustered, as expected, with the MAP kinases. However, in contrast to previous suggestions brought forward before the full complement of mammalian ERKs had been characterised [33,45], they do not specifically cluster with ERK1/2. Rather, they lie outside the cluster of typical MAP kinases comprising the p38, JNK and ERK1/2 classes from human and yeast. Pfmap-2 lies at a basal position relative to the MAPK family, indicating no preferential relatedness to any of its subfamilies. Pfmap-1, in contrast, clearly associates with ERK8, a recently described member of the ERK family which, like Pfmap-1, has a large extension at the C-terminus [46]. In the orthologous rat enzyme ERK7, a similar extension has been shown to be involved in regulation of enzymatic activity [47,48]. It has hence been proposed that ERK8/7 may not be part of typical three-component (MEKK-MEK-MAPK) modules which are the hallmark of the ERK1/2, p38 and JNK pathways. Formal demonstration that ERK8/7 is not regulated by classical MEKs in mammalian cells is difficult because of the numerous MEK homologues present in the genome. The situation in *P. falciparum *therefore provides a first clear example that *in vivo *regulation of a kinase related to ERK8/7 does not require a typical MEK, since no member of the latter family is present in the parasite's genome (see above). It is perhaps unsurprising that *P. falciparum *lacks MAP kinases of the ERK1/ERK2, p38 of JNK subfamilies, given the absence of MAPKKs and STE-like MAPKKKs in the genome. In summary, our data indicate that although the malaria parasite uses MAPK homologues, these are not part of three-component modules – to our knowledge, *P. falciparum *is the first eukaryote demonstrated to lack such modules.

#### Cell cycle control kinases

Three *P. falciparum *kinases cluster with the cell division kinase group that includes the human cell cycle CDKs. PfPK5 [MAL13P1.279] appears orthologous to yeast cdc28 and to human CDK1-3. PfPK5 displays similar levels (60% identity) of overall homology to both mammalian CDK1 and CDK5; however, in our analysis this enzyme clearly clusters with the former, lending support to the idea that this enzyme is a functional homologue of the major cell cycle control kinases, as previously suggested [49,50]. The other two malarial enzymes that clearly cluster within the CDK group, Pfcrk-3 [PFD0740w] and Pfcrk-1 [PFD0865c], cannot be assigned an orthology with any yeast kinase. However, Pfcrk-1 appears to be related to human CDKs such as CDK10 and CDK11 that are involved in transcriptional control, consistent with earlier reports [51] that this enzyme shares primary structure features with the human PITSLRE (CDK11) kinases. Pfmrk [PFL00141] was initially described [52] as a putative homologue of the CDK-activating kinases (CAKs) such as mammalian CDK7, and subsequently shown to be able to undergo some activation by human cyclin H (the cognate cyclin activator of mammalian CDK7) and by Pfcyc-1, a *P. falciparum *protein with maximal homology to cyclin H [50,53]. However, in our tree Pfmrk appears not to be included in the CDK7 cluster, but instead lies at an intermediate position between the MAPK and the CDK groups. It is relevant to mention here that sequence-based prediction of kinase-cyclin pairs is difficult: for example, PfPK5, a clear CDK1-3 orthologue, is unexpectedly activated very efficiently *in vitro *by human cyclin H (a CDK7 activator) and p25 (a highly specific CDK5 activator), among other cyclin-related proteins [50]. This may be explained by structural properties making this enzyme very prone to adopt the active conformation [54]. Extreme caution must therefore be exercised in predicting precise functions for the four cyclin-related proteins which have been identified so far [55].

The positions of the clusters containing (i) PfPK6 [PF130206] [35] and Pfcrk-5 [MAL6P1.271], and (ii) Pfcrk-4 and uncharacterized MAL13P1.196, are consistent with the data in the general tree, and confirm the previously detected relatedness of two of these enzymes to both CDKs and MAPKs. Overall, the number of clear orthologues of cell division kinases in the *P. falciparum *genome is smaller than that in the yeast or human genomes, and may represent a minimum complement of such kinases that are necessary for the completion of a eukaryotic cell cycle. Alternatively, some cell cycle control functions assured by CDKs in human cells may be taken over, in *Plasmodium*, by some of the CMGC kinases with no clear relatedness to established families.

#### Other CMGC kinases

A number of CMGC group kinases interact with factors involved in mRNA splicing. PF11_0156 clearly is an orthologue of human PRP4, a kinase that is associated with mRNA splicing and histone deacetylation and that is conserved in most eukaryotic genomes (including *Schizosaccharomyces pombe*, but not *Saccharomyces cerevisiae*) [56,57]. Human SRPK1 phosphorylates the "Serine-Arginine-rich pre-mRNA splicing factors" called SR proteins, and homologues are conserved in all eukaryotic genomes [58,59]. Two *P. falciparum *kinases (PFC0105w and PFl4_0408) cluster with SPRK. Both these kinases have an insertion between domains VIb and VII that is a distinctive feature of SRPKs. Previously described PfLAMMER [PF14_0431] [38] associates with yeast kns1 [60] and the related human LAMMER kinases CLK1-4 that also phosphorylate SR proteins [61].

Other kinases clustering within the CMGC group include a single orthologue of casein kinase 2α [PF11_0096]. Other eukaryotes have at least 2 alpha subunit-encoding genes, emphasizing the relative simplicity of the *P. falciparum *kinome. As detected on the general tree (Fig. 2), three malarial enzyme cluster with the GSK3 family, the most closely related to human GSK3α/β being the recently characterised PfGSK3 [PFC0525c], which appears to be exported into the host erythrocyte [37].

In several instances our phylogenetic classification of individual kinases differs from the previously reported classification based on BLAST searches. There are at least two reasons for this discrepancy. Firstly, our analysis is based on a comprehensive catalogue of protein kinases from *P. falciparum*, and we have access to comprehensive catalogues from several other organisms. In contrast, several malarial ePKs were classified at the time of their initial identification several years ago, when the sequences could be compared only to non-comprehensive sets. As an example, both *P. falciparum *MAPKs were identified before the mammalian ERK8/7 enzymes were discovered, and the closest sequences available at the time were those of the ERK1/2 family. Secondly, it has been reported that BLAST performs poorly in assigning orthology between human and *C. elegans *genes [62]. This is because of extensive independent gene duplication on the lineages leading to the two organisms. Humans and *P. falciparum *are much more distantly related and there has been extensive gene duplication on the human side. Our data support the view that reliable assignments of orthology between genes in distantly related species might only be assigned through the construction of phylogenetic trees and suggest that comparisons based on BLAST must be interpreted cautiously.

### FIKK, a novel, *Apicomplexa*-specific group of ePK-related proteins

That only 65 typical ePKs were identified in this search is somewhat surprising, as *Saccharomyces cerevisiae*, whose genome (12 megabases) is half the size of the *P. falciparum *genome (24.8 megabases), encodes approximately twice as many enzymes of this family.

**Figure 4:**
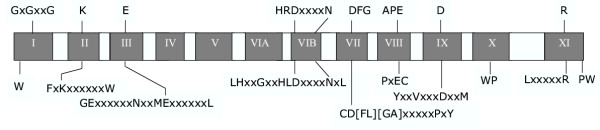
**Comparative primary structure of FIKKs and typical ePKs. **The eleven subdomains of the protein kinase catalytic domain are indicated in the central bar. The residues which are conserved in most ePKs (see legend to Table 1 for details) are indicated at the top. The corresponding residues in FIKKs are indicated under the bar, together with some of the motifs with which they are associated and which are conserved in all FIKK family members.

In preliminary analyses, 21 sequences identified in the HMM search appeared to form a tight cluster that is relatively distantly related to the more typical ePK groups discussed above. Based on an amino acid motif corresponding to subdomain II of the ePK catalytic domain, and which is well conserved in members of this novel family, we called this group "FIKK". In addition to the ePK catalytic domain-like region, the FIKK sequences all have a highly variable N-terminal extension, and in some cases the catalytic domain itself is interrupted by large insertions (as is the case for several of the 65 "typical" malarial ePKs, see below). An alignment of the FIKK kinase-like domain with the 65 typical ePKs in the *P. falciparum *genome showed that they share most of the residues that are conserved in the ePK catalytic domain. Indeed, with the exception of the Glycine triad in subdomain I, all residues which are crucial for phosphotransfer or structural stability of protein kinases, and therefore well conserved throughout the family, are present in all members of this family (see Table 1 and Fig. 4). In contrast, no FIKK sequence possesses a full Glycine triad (GxGxxG) in subdomain I. This triad is present in a majority of ePKs and is involved in positioning the ATP molecule in the catalytic cleft [63]. However, one, and sometimes two glycine residues are present in subdomain I of the FIKK sequences. This is also the case in a number of enzymes with demonstrated protein kinase activity from many organisms (including *P. falciparum*) [40], and it is clearly established that ATP binding and phosphotransfer ability is not dependent on the presence of a Glycine triad. Although lacking the Glycine triad, all FIKK sequences possess an N-terminal extension, with a conserved tryptophan residue in the region that corresponds to subdomain I. One of the FIKK sequences is represented in PlasmoDB as two contiguous ORFs (PF14_0733 and PF14_0734) separated by a gap. This is presumably due to erroneous prediction: alignment with other FIKKs clearly shows these sequences represent two parts of a single member of the FIKK family rather than two separate genes. Furthermore, RT-PCR across the two predicted ORFs demonstrates that both sections are present on the same mRNA molecule. Interestingly, sequencing of the RT-PCR product showed that the open reading frame in the cDNA is interrupted by an in-frame stop codon, which is presumably the cause of the misprediction of the gene structure. That this sequence is cDNA than genomic is ascertained by the presence of an intron near the 3'end (see Additional file 2). Whether PF14_0733/4 is a transcribed pseudogene, or whether a protein can be produced by readthrough of the internal stop codon as has been documented for another *P. falciparum *gene [64], remains to be determined. In any case, it appears there are only 20 FIKK sequences in the genome, instead of the 21 that were counted originally (see above).

**Table 1:** Variability in key residues of the protein kinase catalytic domain. The residues indicated at the top are: G1, G2, G3, the three residues constituting the glycine triad (corresponding to G51, 53 and G56 in human PKAα), and which form a hairpin enclosing part of the ATP molecule; the lysine in subdomain II (K73), which contacts the α- and β-phosphate of ATP, anchoring and orienting the ATP; the glutamate of subdomain III (E92), which forms a salt bridge with the former residue; the aspartate and asparagine within the HRDXXXXN signature motif of ePKs in subdomain VIB (D167, N172), the former of which is thought to be the catalytic residue acting as a base acceptor; the aspartate in the DFG motif of subdomain VII (D185), which binds to the Mg^2+ ^(or Mn^2+^) ion associated with the β-and γ-phosphates of ATP; the glutamate in subdomain VIII (E209), which forms a salt bond with the arginine in subdomain XI and provides structural stability of the C-terminal lobe; and the aspartate in subdomain IX (D221), which is involved in structural stability of the catalytic loop of subdomain VI through hydrogen bonding with the backbone. The conservation status of these residues in the 65 malarial typical ePKs is summarized at the top of the Table, and that of the 20 FIKK family members is presented at the bottom. It is immediately apparent that with the exception of the Glycine triad in subdomain I, all important residues are extremely well conserved in the FIKK sequences

Residue	G1	G2	G3	K	E	D	N	D(FG)	E	D	R
subdomain	I	I	I	II	III	VIB	VIB	VII	VIII	IX	XI

**"Typical" ePKs (65)**											
Number not conserved	16	10	27	0	4	0	0	1	5	2	2
% conserved	75	85	58	100	94	100	100	98	92	97	97
Amino-acid substitution			11S 3A		I MAL7P1_18 N PF14_0408 N PFA0380A K PFB0665w			K PFB0665w	Q MAL6P1_108 Y PF11_0060 Y PF11_0220 N PF11_0377 Q PFC0420w	E PF11_0220 L PFI1415w	N MAL7P1-18 N PF10_0160
Lacking all three Gs in subdomain I	MAL7P1-18 MAL7P1_73 MAL7P1_91 PF11_0060 PF14_0408 PFA0380w PFI1415w PFL0080c										
**FIKK**											
Number not conserved	13	12	17	0(a)	0	0	0	0	0	1	0
									
	All 20 FIKK have a conserved W in a [ILV][YF]W[NTS]XX[GC] motif approx 100 residues upstream of the FIKK motif									E PF14_0733	
% conserved	28	33	5	100	100	100	100	100	100	95	100

Note: a. This residue corresponds to the first K in the FIKK motif.

**Figure 5:**
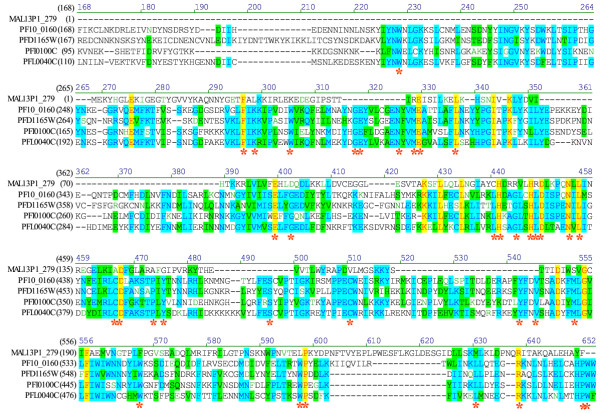
**Alignment of four representative sequences of the FIKK family with a typical ePK **(PfPK5 [MAL13P1.279], a CDK homologue). Asterisks indicate those residues that are invariant in all 20 FIKK sequences.

**Figure 6:**
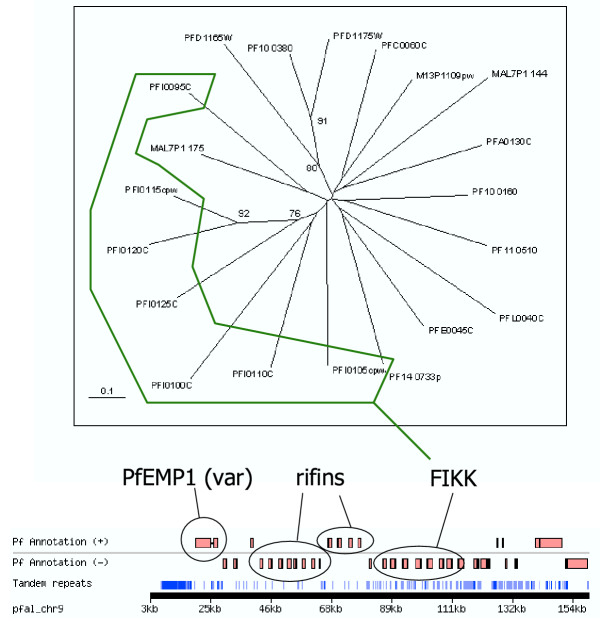
**A tree of the FIKK family. **Phylogenetic tree of FIKKs from *P. falciparum*. The tree was compiled using conserved portions of aligned sequences (see Additional file 3) using a protein distance matrix method. The scale bar represents 0.1 mutational changes per residues (10 PAM units). Bootstrap values over 75 are shown. The bottom panel shows a map of one of the telomeric and subtelomeric regions of chromosome 9 obtained from the PlasmoDB website. The location of genes encoding proteins of the var/PfEMP1 (Duffy et al., 2003), rifin (Kyes et al., 1999) and FIKK (this study) families is indicated.

In addition to the residues conserved in typical ePKs, several amino-acid motifs are fully conserved in all members of the FIKK family (Fig. 4 and 5). These can be used to define signature motifs, which allowed us to perform a number of motif searches in various databases, to determine whether members of this ePK-like family are present in other organisms. Interestingly, sequences containing such motifs could be retrieved only from *Apicomplexan *species: 20 sequences in the *P. falciparum *genome, one in *P. berghei *(Pb75h08p1c-3-1074-4583), one in *P. yoelii *(chrPy1 00951-1-3319-5523), one in *P. knowlesi *(Pk2145b11q1c-4-8079-3688) and one in *P. vivax *(Pv402596-4-9942-5746). In contrast, no FIKK family member was found in the (yet incompletely sequenced) genomes of *P. chabaudi *or *P. reichenowi*. Searches of the NRprot database, which contained sequences representing all eukaryotic and prokaryotic phyla, yielded only the *Plasmodium *sequences mentioned above (20 in *P. falciparum*, and one each in *P. berghei*, *yoelii*, *knowlesi *and *vivax*). In agreement with the motif searches, BLAST analysis of the NRprot database with PF10_0160 finds 20 *Plasmodium falciparum *and one *yoelii *sequences among the top hits (E < 10^-37^). Weaker hits (E > 10^-5^) are mostly MAPKs from a variety of organisms. Further investigations using Apicomplexan genome project databases (Sanger and TIGR) allowed us to identify one such sequence in *Toxoplasma gondii *and one in *Cryptosporidium parvum*. Taken together, these data strongly suggest that the FIKK group is specific to Apicomplexa, and has undergone a dramatic expansion in *P. falciparum*. Interestingly, of the 20 FIKK sequences in the *P. falciparum *genome, 7 are located on chromosome 9, where they are arranged in a contiguous subtelomeric tandem array, a common location for genes involved in antigenic variation such as those of the *var*/PfEMP1 [65] or Rifin [66] families (Fig. 6). On the tree depicted in Fig. 6, these sequences (PFI0095c to PFI0125c) tend to cluster together. The structure of the tree (no major subgroups, with most of the branch points very close to each other and a fairly uniform branch length) suggests a rapid and presumably recent expansion of the family. This hypothesis is supported by the presence of the tandem array, an indicator of gene duplication. Furthermore, the presence of only one FIKK gene in several other apicomplexan species is consistent with the expansion in *P. falciparum *being a recent event. Obviously, a definite conclusion about the species distribution of this gene family will have to await the completion of additional genome sequencing projects, especially with respect to other *Plasmodium *species and other Apicomplexan genera.

Although no experimental evidence is available that associates PK activity with any of the FIKK sequences, the fact that all residues required for phosphotransfer and ePK folding are present strongly suggests that these proteins are indeed protein kinases. Some FIKKs have a predicted signal peptide (PFD1165w, PFE0045c, MAL13P1.109, PFI0095c, PFI0105c, PFI0110c) and/or transmembrane helix (PFD1165w, PFD1175w, PF10_0160, PFI0110c, PFI0125c, PFI0100c has two) at the N-terminus. Otherwise, aside from their similarity to the kinase domain, no recognised Pfam domains are found in these proteins. Two of the FIKK sequences have been identified as *P. falciparum *antigens in the context of immunological studies: the R45 trophozoite antigen (PFD1175w) [67] and the 3.8 protein (PF10_0160). No function has previously been attributed to either of these proteins. R45 has a large insertion of 570 residues, comprising mostly His, Lys, Asn, Ser and Asp residues, relative to the other members of the FIKK family. The belonging of R45 to a 20-sequence family in the *P. falciparum *genome has been discovered independently in the context of research into the R45 antigen (Schneider and Puijalon, personal communication, to be published elsewhere).

### Features of gene structure

Table 1 presents the degree of conservation, in malarial ePKs, of residues that play a crucial role in ePK enzyme function (see legend to Table 1 for details). As is the case in ePKs from other eukaryotes, the Glycine triad is not complete in many PfPKs and in all FIKKs, and none of the three glycine residues are present in 8 of the 65 "typical" ePfPKs. Other important residues are better conserved in the malarial PKs. The observation that some sequences (e.g. PF11_0060, PF14_0733 and MAL7P1.18) lack more than one of these conserved residues raises the question of their ability to function as protein kinases. These may represent kinase-dead scaffold proteins similar to those found in other eukaryotes, such as KSR [68]. In contrast, all 20 FIKKs possess essentially all these residues, despite a conservative D > E substitution in subdomain IX of PF14_0733.

Like in many other plasmodial proteins, large extensions rich in charged and/or polar residues, and in some cases repeated amino acid motifs, are found adjacent to the catalytic domain of several PfPKs. Several enzymes also carry such sequences as insertions within the catalytic domain. The function of these elements is as yet undetermined, although there is evidence in some cases [e.g. Pfmap-1, [32]] that extensions are absent from the enzymes in parasite protein extracts, presumably as a result from proteolytic cleavage. In some sequences (e.g. PFD0740w [Pfcrk-3] and PFC0755c [Pfcrk-4]), large insertions have been mapped to the hinge region between adjacent β-sheets in the N-terminal lobe; hence, it can be argued that such insertions may not interfere with proper folding of the catalytic domain (Equinet and Doerig, unpublished).

### Organelle targeting

**Figure 7:**
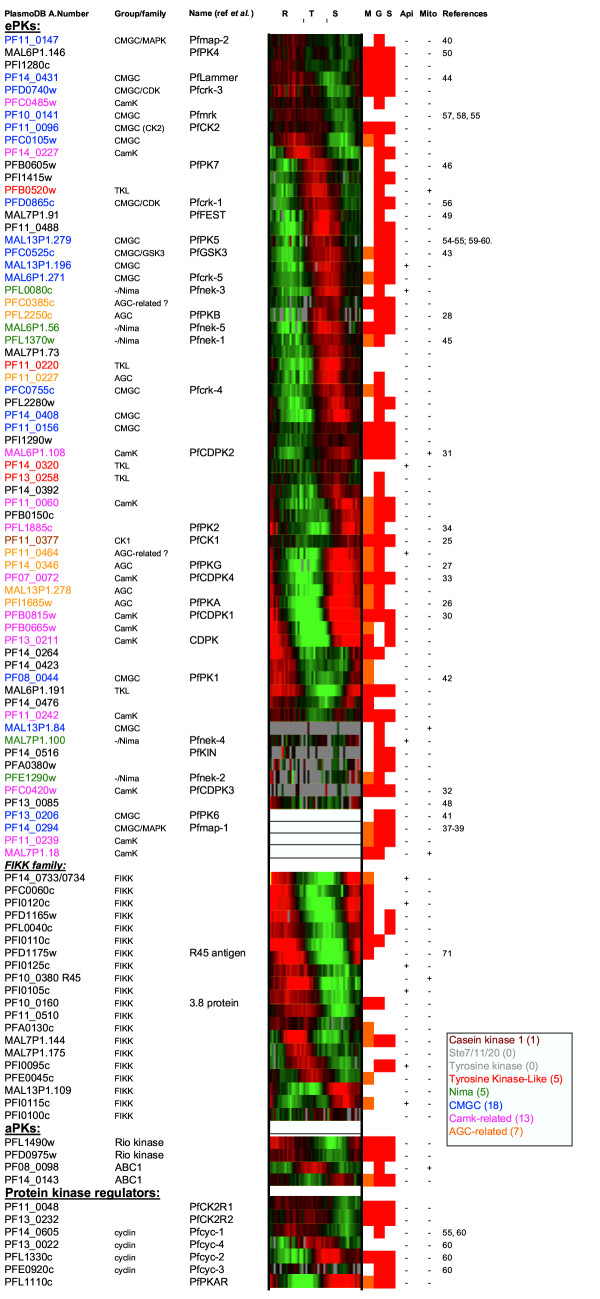
***P. falciparum *ePKs and related proteins, and stage-specificity of their expression. **PlasmoDB gene identifiers are indicated in the left column, followed by the published names where applicable. Identifiers of enzymes belonging to defined ePK groups appear in color (see the inset for color codes). Microarray data from the Le Roch et al. and Bozdech et al. studies available on PlasmoDB, were compiled to produce the third column. Genes were arranged in function of the timing of their expression according to Bozdech et al., to illustrate the fact essentially all of them are expressed in a regulated way during erythrocytic schizogony, and that this process involves sequential but overlapping expression of most kinases in the genome. The phaseogram (data generated by Bozdech et al. and available on the PlasmoDB website) represent the relative abundance of mRNAs throughout the erythrocytic asexual cycle, measured by two-colour competitive hybridisation between total RNA from each time point and a reference pool of total RNA from all time points (48 time points, i.e. one per hour during the 48 hours of the asexual cycle, starting one hour post invasion). The phaseogram shows the red/green colorimetric representation of the gene expression ratio for each oligonucleotide. Green: negative ratio (no expression), red: positive ratio (expression); grey or white: no data. See Bozdech et al. (2003) and PlasmoDB for details. To the right of the phaseogram, the presence or absence of mRNA in samples from merozoites (M), gametocytes (G) and sporozoites (S) is indicated by red boxes (data generated by Le Roch et al.). Where only one of the two synchronised merozoite population gave a signal, the M box is colored in orange (see Le Roch et al. 2003 for details). Columns to the right indicate those molecules which, according to the gene prediction algorithm used in PlasmoDB, possess a putative apicoplast or mitochondrion targeting sequence (see text for details).

Malaria parasites possess two organelles with extra-chromosomal DNA: the apicoplast and the mitochondrion. The apicoplast is a four-membrane organelle carrying a circular 35 kb DNA whose structure is very similar to that of plastid genomes. It is specific to the Apicomplexa (hence its name), and thought to originate from secondary endosymbiosis [69]. As is the case for chloroplasts in plants [70], it appears that many genes whose products are essential for apicoplast function and survival have been transferred to the "host cell" nucleus; products of these genes must be addressed back to the organelle. A bipartite peptide has been identified and shown to be necessary and sufficient for targeting a protein to the apicoplast [71]. The 35 kb genome of the apicoplast does not encode any PK, but it is to be expected that protein phosphorylation is necessary for function and maintenance of the organelle. We used an algorithm available on PlasmoDB to determine that 5/65 typical ePfPKs (including 2 NIMA-related kinases) and 6/18 FIKKs are predicted to be addressed to the apicoplast (see Fig. 7). Likewise, four kinases (none of them of the FIKK family) possess a potential mitochondrion-targeting signal sequence, as defined by the algorithm available on PlasmoDB [72]. It is important to emphasise that presence or absence of targeting signals relies on gene structure prediction algorithms, which have been demonstrated to be erroneous in some instances (see ref. [55] for an example); therefore this must be considered with caution until the 5'end of the cDNAs has been sequenced, and targeting to the organelle has been verified experimentally by the transfection of constructs expressing GFP-tagged proteins.

### Regulatory subunits

Proteins devoid of kinase activity but which are known to associate with, and regulate the activity of, ePKs have been identified in PlasmoDB. These include four previously characterised cyclins [PF14_0605, PF13_0022, PFL1330c and PFE0920c] which have been demonstrated to associate with histone H1 kinase activities in parasite extracts [50,55], a PKA regulatory subunit [PFL1110c], which as expected is able to down-regulate PKA in parasite extracts (Merckx and Doerig, unpublished), and two putative CK2 regulatory subunits [PF11_0048 and PF13_0232].

### Genes encoding aPKs

BLASTP searches of PlasmoDB were performed using atypical protein kinases (aPKs) from *Homo sapiens *as queries. GeneDB was also used to look for relevant Pfam domains (ABC1, FAT, FATC, Bromodomain, RIO). Two members of the RIO kinase family were found: PFL1490w (RIOK1-like) and PFD0975w (RIOK2-like). Enzymes of this family are involved in rRNA processing in *S. cerevisiae *[73]. We also identified two putative members of the ABC1 family of atypical protein kinases [PF08_0098 and PF14_0143]. Some *P. falciparum *genes (e.g. PFD0685c and PF14_0326) display regions with low-level similarity to the histidine kinase domain (scores between 4 and 5 with Pfam entries PF00512 and PF06580), but the significance of this observation remains to be established.

No significant hits were obtained with A6 kinases, Alpha kinase, pyruvate dehydrogenase kinase, aminoglycoside phosphotransferases or DNA-dependent kinases. In contrast, we identified a malarial phosphatidyl-inositol-3 kinase homologue [PFE0765w], in agreement with experimental studies [74] and the presence of a PKB homologue (see above) demonstrating the presence of a phosphatidyl-inositol pathway in the parasite. However, the PI3K homologue, like two other sequences (PFE0485w and PFD0965w) related to PI4K, appears not to contain the FAT and FATC domains which are present in PIKs from other organism and have been associated with protein kinase activity [75]. Hence, it may be that these three enzymes function solely as phosphatidylinositol kinases, a proposition that requires experimental testing. Overall, these results on malarial aPKs contrast with those obtained from the recently-sequenced *L. major*, *T. brucei *and *T. cruzi *genomes, where ABC1 and RIO kinases were found, as were PIKK (with the FAT and FATC domains), PDHK and Alpha kinase family members (Parsons and Ward, unpublished).

### Expression pattern of PfPKs during the *P. falciparum *life cycle

Data from two studies [76,77] of the *P. falciparum *transcriptome during development are available on the PlasmoDB database. We compiled these data to obtain a general picture of PfePK gene expression during erythrocytic development (Fig. 7). It is clear that the steady-state level of mRNA is developmentally regulated for all the PfPK genes, in accordance with the unique gene expression pattern described in this organism by Bozdech et al. [76]. Most of the PfePKs are expressed in trophozoites and schizonts, but some PK mRNAs are clearly predominantly detected in rings, the younger form following erythrocyte invasion. Data from Le Roch et al. [77] included a transcriptome analysis of additional development stages: free merozoites, gametocytes and sporozoites. Compilation of data from this study indicated that a small number of PfePKs are specific to gametocytes, including two of the NIMA-related kinases (one of which is potentially targeted to the apicoplast), one of the MAPKs (Pfmap-2), and PfKIN, an enzyme previously described as related to the SNF1 family (see above). Gametocyte-specific expression had been described in the literature for the latter two enzymes [42,45]. Overall, and despite some discrepancies, there is good agreement between the two studies with respect to PfePK genes, as illustrated by the observation that PfePKs whose expression is detected in late schizonts and segmenters by Bozdech et al. are also detected in free merozoites by Le Roch et al. At least some of these enzymes are likely to play a role in invasion of the erythrocyte by the merozoite. As expected, the PK genes that are gametocyte-specific according to Le Roch et al. (and hence likely to play a role during sexual development of the parasite) give low intensity signals in the dataset from Bozdech et al. (see for example Pfmap-2 or Pfnek-4 to illustrate this point).

## Conclusion

This study has allowed us to classify the 65 typical ePKs encoded by the *P. falciparum *genome, and to establish the presence of a novel group of ePK-related genes, the FIKK family, which, from analysis of currently available databases, appears to be specific to Apicomplexa and considerably extended in *P. falciparum*. The number of genes encoding protein kinases is somewhat smaller than expected from analogy with other organisms. We cannot exclude that our study, which is based on sequence similarity with ePKs, may have missed genes encoding proteins with protein kinase activity, but with a primary structure that would be too divergent from that of known ePKs to be identified. Nevertheless, it is hoped that the present study will facilitate investigations into the regulation of many pathways and processes operating during growth and development of the parasite. In addition to the FIKKs, several malarial ePKs belong to "orphan" groups, as they do not cluster clearly with established ePK groups as defined in model organisms. Furthermore, our analysis provides evidence that elements which are usually found in eukaryotes are absent or dramatically modified in malaria parasites. Such elements include MAPK pathway components and PKC, for example. These important divergences between the malarial and human kinomes reflects the vast phylogenetic distance between Apicomplexans and Opisthokonta, and strengthen our expectations that specific interference with essential functions of the parasite can be achieved through the use of protein kinase inhibitors.

## Methods

### Identification of ePK genes in the *P. falciparum *genome

The set of predicted peptides of the *Plasmodium falciparum *genome 3D7 [78] was downloaded from PlasmoDB [8]. A Hidden Markov Model search [79] of the predicted proteins encoded by the genome was carried out using a eukaryotic protein kinase profile downloaded from the Pfam database [80]. In addition, PlasmoDB was searched for proteins carrying a Gene Ontology molecular function assignment [81] of 'protein kinase activity' (GO:0004672). This allowed us to constitute an initial list of 108 sequences. After inspection, 15 were removed that had high e-value (>0.01), low HMM scores (<-110) and visibly lacked a protein kinase domain. The remaining 93 sequences were aligned using our own Hidden Markov Model, trained on a complete set of human protein kinases, to check for the presence of the key kinase motifs. In addition, the genomic context of each putative kinase gene was examined to check for missing exons using GeneDB  and Artemis [82]. Eight proteins, the first four of which have a PlasmoDB enzyme assignment to EC2.7.1 (phosphotransferases), lacked sufficient similarity to typical eukaryotic protein kinases to be aligned meaningfully across the kinase domain. These sequences were: PF13_0166, PFC0945w, PFE0170c, PFI1275w, MAL7P1.127, MAL7P1.132, PF11_0079 and PF14_0264; they were removed from further analysis. A further 20 sequences constituted the FIKK family (see below). This set of closely related, but atypical, sequences was analysed separately. The remaining 65 sequences represent the complement of typical protein kinases in *P. falciparum*. Although the Hidden Markov Model used for the alignment is based on an extensive training set, the alignment did require some manual optimisation. This is partly because of the extreme diversity of the gene family and partly because many predicted proteins from *P. falciparum *contain large repetitive insertions (Hidden Markov Model-based alignment protocols would be expected to cope better in these circumstances than other common methods). A full alignment of the kinase domains is shown in Additional file 1. Once a definitive set of the 65 sequences representing typical ePKs had been assembled, a phylogenetic tree was produced using Phylip [83], with the Protdist and Fitch algorithms. Human protein kinases were added to the alignment in order to improve the visualization of the main groups of protein kinases among the *P. falciparum *sequences. Only gap-free conserved regions of the alignment were used for the construction of the tree (164 amino acid positions). Bootstrap values supporting the branches of the tree are rather low; this is to be expected given the diversity of the protein kinase family.

## Authors' contributions

PW performed most of the database searches for ePK-related sequences and constructed the FIKK phylogenetic tree; LE contributed to the searches for aPKs, compiled expression data and performed the *in silico *and *in vitro *analyses of the FIKK family. JP generated the HMM-derived alignments, constructed the ePK phylogenetic trees and contributed significantly to their description in the text. CD coordinated the study and wrote the larger part of the manuscript. All authors read and approved the manuscript.

## Supplementary Material

Additional File 1 Alignment of the 65 "typical" *P. falciparum *ePKs used for constructing the tree in Fig. 2. Please see the Methods section for details on how the alignment was generated. Click here for file

Additional File 2 partial sequence of the cDNA for the gene PF14_0733/PF14_0734. Click here for file

Additional File 3 Alignement of the 20 FIKK sequences used to construct the tree in Fig. 6. Click here for file
