# Supplementary material for: Protein kinases of the human malaria parasite Plasmodium falciparum: the kinome of a divergent eukaryote
Source: BMC Genomics. 2004 Oct 12;5:79. doi: 10.1186/1471-2164-5-79 (PMC526369; doi:10.1186/1471-2164-5-79)
Supplement: Additional File 2 — partial sequence of the cDNA for the gene PF14_0733/PF14_0734. [file 1471-2164-5-79-S2.doc]

TGGCTAGATATCGAGGGGAATATTTAGTAAATGCTGAAAATTTTGTAATGGAAGCTGTCGCTTCTGCTTTTTTAACGGAATATCATCCAGGAATAACACCAAAATTATATAAAATATTATATGATCCGATT**TGAGAAAATAAAAAGAGTTTACATAAAATAGCTTTTAATGATTTAGGTGCATTTAATTATATTTTGCGTAATAGATTAAAAAGTAACATTGAAGGAAATATTGTAATAATTTCTGAATTATATGGTCAAGATATATTTAATTATATTGATAAAAAACGACTAGATATTGGTA**TGGATGATGATGATGATGATTTAGTTTTAACTGTTGAAGAAAAAAAGAGTATTCTTTATAAAGCTTTAAATTTATATACAAGATTACATGAAGCAGGTTTAGCACATCTAGATTTATCAGCAGAAAATGTTTTAATCGATGAGAATAATGAGGTACGTTTATGTGATTTAGGTAAAAGTACACCTGTGTATACTACTAGCTTAAGACATTTAGATGACAGTTTAGATTTAGCAATTTTTGAATCCTGCGTACCATGTGTAGGCAAAGAAGCTTATATGCCTCCTGAGTGTATGAAGTTATATAAAGAATATCGTAAAATGAAGATAAGTAGTCCCTTTGATTATGCTAATTCTGTAAGGGATAGAAGAGAAAGAAGAAAATGGTATTTTGATGTTTTAACAGCTGAAAAATATATGCTTGGAATTTTCTTTATGTGGATCTGGAATGAAGGCCATTTATGGGATTGTTCAGATCCATCAAAAGATGAAATTTTTAATGAAATAAATGAATGTGAAATGGACTTGGATAAGTGCGATTTAACTGATAATTGGCCTGAAGGGTTGAAAGCCATGATTA**AGGTAAATGGATTTGATTATATATATATATATATATATATATATATATATATATATATATATATATATGTATGTATATATGTATGTATGTATGTATATATGTATATATGTATATATATGTATATCTATCTATATATATATATATAAATATAATAATTTTTTTTTTATTTTTTTTTTTTTTTTTTTTTTTTATAGA**GATTATTAAATTTTGAATCTAGGAAGGAATTAAATCTAAAGGATATATATGAC

Additional file 2: partial sequence of the cDNA for the gene PF14_0733/PF14_0734. The internal stop codon is highlighted in red. Green underlined characters indicate the gap between the PF14_0733 and PF14_0734 open reading frames. Clearly, this part of the sequence is present in the cDNA. Blue characters indicate the intron that was detected experimentally, which is larger than that predicted in PlasmoDB (the predicted intron is underlined).
